# Women’s empowerment and nutritional status of children: new evidence for Bangladesh

**DOI:** 10.1017/S1368980026101980

**Published:** 2026-02-18

**Authors:** Saira Parveen Jolly, Jurjen van der Schans, Robert Lensink, Kaosar Afsana, Md Atiqul Islam, Regien Biesma

**Affiliations:** 1 Global Health Unit, Department of Health Sciences, University Medical Center Groningen, Groningen, The Netherlands; 2 BRAC James P Grant School of Public Health, BRAC University, Dhaka, Bangladesh; 3 Department of Economics, Econometrics and Finance, University of Groningen, Groningen, The Netherlands; 4 Department of Statistics, Jagannath University, Dhaka, Bangladesh; 5 Julius Center Global Health, University Medical Center Utrecht, University of Utrecht, Utrecht, The Netherlands

**Keywords:** Women’s empowerment, Survey-Based Women’s Empowerment Index, Under-five children, Nutritional status, Food intake, Acute respiratory tract infection, Bangladesh

## Abstract

**Objectives:**

This study examines aspects of women’s empowerment related to the nutritional status of under-five children in Bangladesh, including their age-appropriate food intake and access to healthcare during acute respiratory tract infection (ARI).

**Design:**

Three waves of the Bangladesh Demographic Health Survey (BDHS) data (2011, 2014 and 2017–2018) were pulled and utilised to construct three domains of the Survey-Based Women’s Empowerment Index, such as social independence, intrinsic agency and instrumental agency. The height-for-age Z (HAZ), weight-for-age Z (WAZ) and weight-for-height Z (WHZ) scores were used to measure the nutritional status of offspring. Two variables were generated to measure age-appropriate food intake and treatment-seeking from medically trained providers (MTP) at the commencement of ARI. Generalised structural equation modelling was performed to develop pathways between women’s empowerment and children’s nutritional status.

**Settings:**

Data were collected from eight administrative divisions in Bangladesh.

**Participants:**

A total of 18 706 married women aged 15–45 years residing with their husbands and having at least one under-five child.

**Results:**

Women’s social independence was positively associated with HAZ (0·25 (95 % CI: 0·22, 0·28)), WAZ (0·21 (0·18, 0·24)) and WHZ (0·06 (0·02, 0·09)). Intrinsic agency positively influenced HAZ (0·03 (0·02, 0·04)) and WAZ (0·02 (0·01, 0·02)). Both social independence and intrinsic agency promoted appropriate feeding, while instrumental agency had a negative effect on food consumption (–0·0026 (–0·005, –0·0002)). Both age-appropriate food intake and seeking treatment from MTP during recent ARI episodes improved nutritional outcomes of offspring.

**Conclusion:**

Maternal social independence and intrinsic agency enhance the nutritional status, food consumption and healthcare access of offspring in Bangladesh.

Undernutrition among under-five children remains a global crisis, affecting 149·2 million children with stunting and contributing to 45 % of deaths in this age group^(1)^. This underscores the slow progress towards achieving Sustainable Development Goal (SDG) 2.2^(1)^. To meet this target, it is essential to integrate various SDG, as improving nutrition is linked to multiple underlying factors. According to UNICEF’s conceptual framework on undernutrition, ensuring access to a safe and adequate diet (SDG 2.1) and quality essential healthcare services (SDG 3.8) can directly address undernutrition^(2)^. Improved nutrition could also prevent up to 75 % of deaths among under-five children due to undernutrition and infectious diseases, further supporting SDG 3.2^(3)^. Children affected by infectious diseases, such as acute respiratory tract infection (ARI), are often prone to various forms of undernutrition and have a high mortality rate^(4)^. This elevated mortality is partly due to limited access to preventive treatments and interventions^(5,6)^. Additionally, empowering women is a key aspect of SDG 5 which can enhance their access to food security and healthcare^(7,8)^. Therefore, promoting women’s empowerment is vital for improving the nutrition and health outcomes of offspring.

Women’s empowerment is defined as the process through which women gain the ability to make strategic life choices in contexts where they were previously denied this power. It involves utilising resources, opportunities and knowledge to make intentional decisions^(9)^, enabling individuals to act on and improve issues important to their lives, communities and society^(10)^. Additionally, it enhances women’s self-esteem, control over material assets, intellectual resources and ideologies through fostering critical awareness in areas like education and healthcare^(11)^.

In the context of Bangladesh, women’s empowerment is significantly affected by cultural and traditional norms, especially patriarchy and purdah. Here, men dominate women through family, marriage and inheritance, limiting women’s freedom of choice, decision-making power and access to resources. These patriarchal norms in Bangladesh confine women to domestic roles and childcare, while men handle financial support and asset management, hindering women’s equality^(12)^. Purdah, involving veiling and segregation, restricts women’s freedom, mobility, education, healthcare and societal participation^(13)^. Additionally, undervaluation, early marriage, practice of dowry, divorce rights, sex bias, birth registration, political awareness, intimate partner violence (IPV) and Shariah law further impede women’s development in Bangladesh^(12,13)^.

Research shows that married women in Bangladesh are more involved in household decision-making related to health and family planning than in decisions about household expenditures and personal autonomy^(14)^. In Bangladesh, women have made progress, partly due to advancements in education and participation in microcredit programmes^(14)^. Despite these advancements, women’s mobility remains limited, with only 13 % of them are visiting health facilities or friends without any permission^(14)^. Evidence shows that addressing women’s decision-making can improve homestead food production^(15)^, dietary diversity^(16)^, the nutritional status of under-five children^(17,18)^ and better access to healthcare during ARI episodes in their children^(19)^. These studies highlight the pathways between food and nutrition security outcomes with women’s autonomy of decision-making. Besides, women’s self-esteem, education empowerment, agency, control over resources and engagement in credit are linked to food and nutrition security in different contexts^(15,16,18,20)^. However, there is a lack of evidence within the context of Bangladesh regarding which aspects of women’s empowerment are the most effective and should be prioritised for policy implementation aimed at empowering women and improving the nutritional status of under-five children.

In Bangladesh, the majority of deaths among offspring are caused by ARI^(21)^. This illness has a harmful effect on the weight of these children^(22)^. Children with ARI are also more likely to suffer from anaemia, stunting and wasting, which increases their risk of death due to treatment failure^(4,23)^. Although the government of Bangladesh has implemented the WHO-recommended Integrated Management of Childhood Illness (IMCI) strategy for treating ARI among under-five children, only 45 % of those affected receive care from an appropriate healthcare provider and 65 % are given antibiotics^(21,24)^. Evidence also shows that parental awareness, care-seeking behaviours and antibiotic coverage were lower among deceased children^(21)^. Furthermore, women often had to obtain permission from their husbands to access healthcare for their child’s ARI treatment^(25)^. This indicates that lack of knowledge and women’s limited autonomy in decision-making hinder access to appropriate care.

We hypothesised that different aspects of women’s empowerment might improve the nutritional status of under-five children. Furthermore, we postulate that these aspects would enhance women’s autonomy in accessing age-appropriate diets for their children and seeking treatment from medically trained providers (MTP) for their offspring experiencing ARI. Unlike UNICEF’s conceptual framework^(2)^, our hypothesis would establish a pathway that appropriate management of ARI can halt the deterioration of nutritional status. It will also examine the indirect effect of the domains of the Survey-Based Women’s Empowerment Index (SWPER) on the child’s nutritional status to explore whether and how age-appropriate food intake and healthcare service utilisation during ARI mediate this correlation. This study will contribute to the literature by exploring the linkages between distinct domains of women’s empowerment and the nutritional well-being of under-five children in Bangladesh.

## Methods

### Study design and data source

We conducted a secondary analysis of data from three waves of the Bangladesh Demographic and Health Survey (BDHS), specifically from 2011, 2014 and 2017–2018. Our study focused on married women with their reproductive age of 15–49 years, currently staying with their husbands whose decision-making capacity might have been dominated by their husbands and having at least one child aged 0–59 months.

### Measures

The exposures of interest were the three domains of the SWPER global^(26)^, while the indicators of nutritional status of under-five children, such as height-for-age Z (HAZ), weight-for-age Z (WAZ) and weight-for-height Z (WHZ) scores, were the outcomes of interest. The age-appropriate food consumption and seeking treatment from MTP at the commencement of ARI were the mediating factors within this research framework.

### Survey-Based Women’s Empowerment Index

We assessed women’s empowerment using SWPER global, which consisted of three domains^(26)^. The first domain, social independence, included six factors: reading newspapers or magazines, women’s educational attainment, age at first childbirth, age at initial cohabitation, the age difference between the husband and wife and differences in educational levels between the husband and wife^(26)^. The second domain, intrinsic agency, was constructed from five factors that assessed women’s attitudes towards wife-beating by their husbands. These factors considered whether wife-beating was justified if the wife went out without informing her husband, neglected her children, argued with her husband, refused sexual relations with her husband and burned food^(26)^. Intrinsic agency, often referred to as ‘power within’, reflects a woman’s internal sense of empowerment. It is the process through which women develop critical awareness of their own aspirations, capabilities and rights^(26)^. This form of agency can be interpreted as both a personal asset and an opportunity, especially in relation to environmental safety, serving as a proxy of their ability to act on personal choices^(26)^. The DHS includes five attitude-related questions on the justification of IPV, which have been widely used in prior studies (including SWPER global) as a proxy for intrinsic agency^(26)^. A woman’s rejection of IPV in these items has been interpreted as a reflection of self-worth, personal dignity, personal safety and awareness of one’s rights, making it a useful indicator of intrinsic agency within the constraints of existing survey tools. Furthermore, a stronger sense of intrinsic agency is typically associated with the rejection of justifications for IPV, indicating greater self-respect and human rights violation^(26)^. Finally, the third domain, instrumental agency, was derived from three factors related to women’s decision-making autonomy, encompassing decisions about their own healthcare service utilisation, substantial household purchases and visits to family and relatives^(26)^.

The SWPER was initially validated for thirty-five African countries and later extended to sixty-two low- and middle-income countries, including Bangladesh^(26)^. For external validation, the index was compared with the Gender Development Index and the Gender Inequality Index; both indices reflect progress towards SDG 5 and showed strong correlations with country-level rankings^(26)^. Although women’s empowerment is context-specific, both South Asia and sub-Saharan Africa share deeply rooted patriarchal norms despite their distinct sociocultural contexts. Notably, both regions demonstrated lower mean scores across all three empowerment domains^(26)^.

### Dietary intake of under-five children

Dietary intake guidelines for offspring are tailored according to their specific age groups. This study followed the dietary guidelines for Bangladeshi children as outlined elsewhere^(24)^. For infants aged 0–5 months, exclusive breast-feeding is recommended as an ideal food source. Breastfed infants aged 6–8 months should be fed two meals daily, each including foods from four of the seven food groups. Breastfed children aged 9–23 months are advised to have three meals a day, incorporating items from four of the seven food groups. Non-breastfed children aged 6–23 months should have three meals daily, ensuring their plates include items from at least four food groups, including dairy products. For children aged 24–59 months, it is recommended to have food from at least four food groups within the last 24 h of interview^(24)^.

### Acute respiratory tract infection and medically trained providers

ARI among children aged 0–59 months was identified by counting living children who exhibited symptoms such as short, rapid breathing and/or difficulty breathing related to the chest within the 2 weeks preceding the survey^(24)^. Additionally, an MTP includes medical professionals such as MBBS doctors, nurses, midwives, Family Welfare Assistants (FWA), Medical Assistants (MA), Sub-Assistant Community Medical Officers (SACMO) and Health Assistants (HA) who work at various levels of the healthcare system in Bangladesh^(24)^. These professionals are trained in the IMCI protocol for ARI.

### Nutritional status of the under-five children

We assessed the nutritional status of under-five children using the HAZ, WAZ and WHZ scores available in the BDHS dataset. All three variables displayed a normal distribution. We excluded observations with HAZ, WAZ and WHZ scores below –6 sd or above +6 sd as these values are flagged as having invalid data and those with missing observations^(27)^.

### Statistical analysis

#### Descriptive analysis

Initially, the continuous HAZ, WAZ and WHZ scores of under-five children were categorised into two groups: stunting and non-stunting, underweight and non-underweight, and wasting and non-wasting. This classification was based on whether the children fell below 2 sd from the median of the WHO Child Growth Standards reference population^(28)^. A composite variable for age-appropriate food intake of offspring was also created, categorised into two groups based on adherence to exclusive breast-feeding, meal frequency and dietary diversity according to age during the 24 h preceding the interview^(24)^. A dichotomous variable was generated for the children suffering from ARI, and another composite variable was generated for the children who received treatment from MTP against ARI within the 2 weeks preceding the interview. The three domains of the SWPER were coded as described elsewhere^(26)^. Later, confirmatory factor analysis was conducted to obtain three latent variables: social independence, intrinsic agency and instrumental agency.

We performed the Pearson chi-square test to determine the association between the nutritional status of offspring and other dichotomous variables. Conversely, for ordinal variables, the Goodman–Kruskal gamma coefficient was employed to gauge the degree of association. The data for categorical variables were presented as percentages (numbers). We conducted Student’s *t* test for symmetrical variables, and the data were presented as mean (sd). Additionally, the Kruskal–Wallis rank test was performed for asymmetrical continuous variables, and the data were presented as median (interquartile range (IQR)). Pairwise correlation was performed to understand the relationship between different factors of social independence, intrinsic and instrumental agency and ARI.

#### Inferential analysis

**Figure 1.**
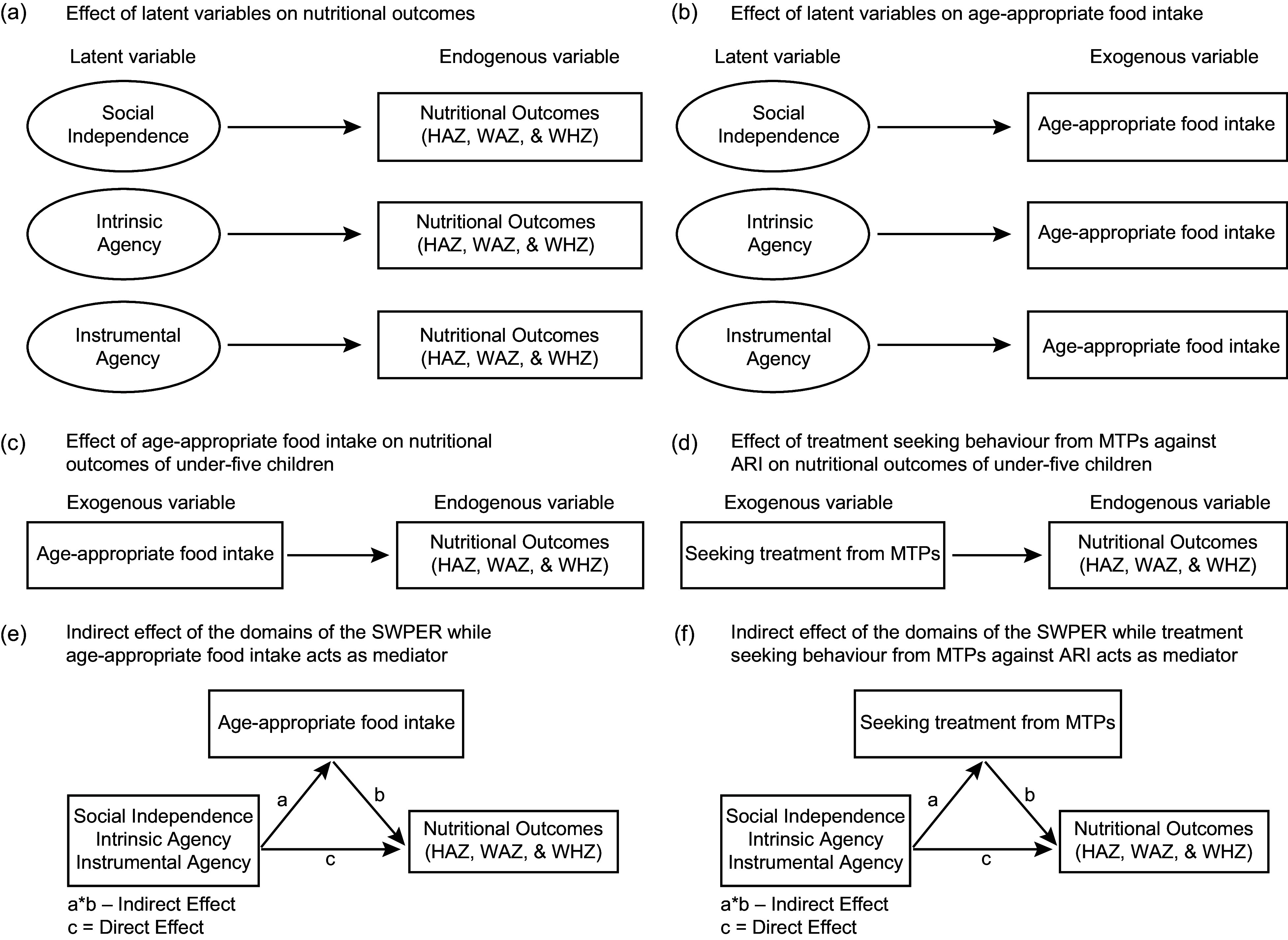
Pathways between latent variables and nutritional outcomes and mediators. HAZ, height-for-age Z score; WAZ, weight-for-age Z score; WHZ, weight-for-height Z score; ARI, acute respiratory tract infection; MTP, medically trained providers.

We performed generalised structural equation modelling (GSEM) to estimate the effect of both latent and exogenous variables on the endogenous variable (Figure 1). We performed GSEM instead of traditional structural equation modelling by integrating several outcome variables (continuous, binary, ordinal, count, etc.) and employing distinct link functions based on the distribution of the outcomes, extending beyond linear regression (Table 1). Moreover, GSEM offers a more adaptable framework for modelling complex relationships between both latent and observable variables. The generalised structural equation model can be expressed as the relationship between latent variables and observed variables:
(1)

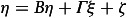




**Table 1.** Categories of the variables and their families and links for conducting the generalised structure equation modelling

Category	Variable	Family	Link
Continuous	HAZ, WAZ, WHZ, women’s year of schooling, age at first birth, age at first cohabitation, age difference between husband and wife, year of education difference between husband and wife	Gaussian	Identity
Binary	Reading newspapers, age-appropriate food intake, ARI, seeking treatment from MTP	Bernoulli	Logit
Ordinal	Beating is justified if wife goes out without telling husband, neglects the children, argues with husband, refuses to have sex with husband, burns the food, and makes decisions on own healthcare, large household purchase and visits to family or relatives	Ordinal	Logit

HAZ, height-for-age Z score; WAZ, weight-for-age Z score; WHZ, weight-for-height Z score; ARI, acute respiratory tract infection; MTP, medically trained providers.

where


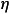
 : vector of endogenous variables (e.g. nutritional status);


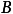
 : matrix of coefficients relating latent endogenous variables;


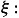
 vector of exogenous latent variables (e.g. food intake, ARI, treatment seeking from MTP against ARI, etc.);


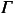
 : matrix of coefficients relating exogenous observed variables to endogenous latent variables;


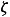
 : vector of structural disturbance terms.

The measurement model for the exogenous latent and endogenous latent variables is given by

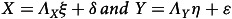




where


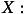
 observed indicators for exogenous latent variables;


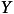
 : observed indicators for endogenous variables;


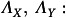
 factor loading matrices;


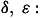
 measurement error vectors, 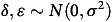 .

The specific latent variables are modelled by
(2)

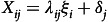




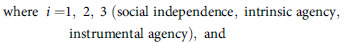




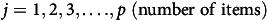




Therefore, the final GSEM for nutritional status is written using equation (1) and (2) as:
(3)

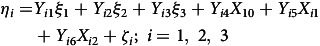




where


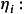
 nutritional status (latent endogenous variable);


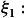
 social independence (latent exogenous);


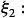
 intrinsic agency (latent exogenous);


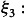
 instrumental agency (latent exogenous);


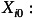
 age-appropriate food intake (observed exogenous);


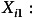
 ARI occurrence (observed exogenous);


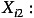
 treatment seeking from MTP (observed exogenous);


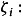
 structural disturbance terms.

Parameters from equation (3) were estimated by the maximum likelihood method depending on the distributional assumptions and their link functions.

For continuous outcomes (in the case of our study, nutritional status), the model is written as:

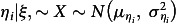




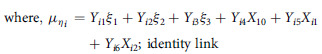




For binary outcomes, the model is written as:

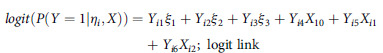




For count outcomes, the model is written as:

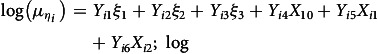




The models’ goodness of fit was assessed using the Akaike information criterion and Bayesian information criterion. Additionally, we conducted mediation analysis to determine the indirect effects of latent variables on the endogenous variables in the presence of other exogenous variables^(29)^ (Figure 1). For this analysis, we calculated the direct, indirect and total effects of the latent variables: social independence, intrinsic agency and instrumental agency on the outcome indicators such as HAZ, WAZ and WHZ and by incorporating age-appropriate diet and seeking treatment from MTP at the onset of ARI as mediators in the models, by using GSEM. Through this analysis, we wanted to assess whether aspects of women’s empowerment were solely contributing to improved nutritional status, or whether they worked together with age-appropriate food intake and treatment from MTP. The basic mediation model analyses the influence of an independent variable 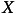 on a dependent variable 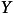 via a mediator 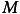 . The mathematical equation of a mediation model is
(i)

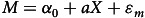




And the outcome model is
(ii)

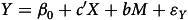




where


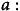
 effect of 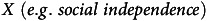 on 
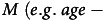
 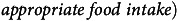 ;


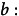
 effect of 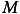 on 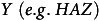 , controlling for 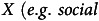 
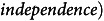
 ;


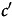
 direct effect of 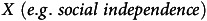 on 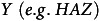 , controlling for 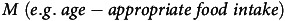 ;


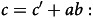
 total effect of 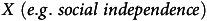 on 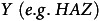 ,

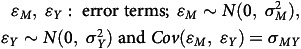




The estimates of all GSEM and mediation analyses were expressed as coefficients (95 % CI). Data analysis was performed using Stata 17.

## Results

### Sociodemographic status of the women

Following the application of our inclusion and exclusion criteria, our analysis comprised a total of 18 706 women. Table 2 presents a comparison of the sociodemographic status of women with undernourished and well-nourished children. The average age of women was comparable between the groups, except for the difference observed between women with wasted and non-wasted children (*P* < 0·05; Table 2). We identified a negative association between children’s stunting, underweight and wasting and the higher educational levels of their mothers and a higher wealth quintile. Additionally, women with undernourished children were more involved in microcredit programmes compared to those with well-nourished children.

**Table 2.** Sociodemographic status of the respondents by nutritional status of their under-five children

Variables	Height-for-age Z score				Direction of difference (B-A)	Weight-for-age Z score				Direction of difference (D-C)	Weight-for-height Z score				Direction of difference (F-E)
	< −2·00 sd (A)		≥ −2·00 sd (B)			< −2·00 sd (C)		≥ −2·00 sd (D)			< −2·00 sd (E)		≥ −2·00 sd (F)		
*n*	5658		13 048			6856		11 850			2163		16 543		
	Mean	sd	Mean	sd		Mean	sd	Mean	sd		Mean	sd	Mean	sd	
Age of the mother, in years, Mean (sd)*	25·89	6·0	25·71	6·0	−0·18^a^	25·99	6·0	25·64	6·0	−0·35^a^	**25·92**	**6·0**	**25·75**	**6·0** ^**b**^	**−0·17**
	%	*n*	%	*n*		%	*n*	%	*n*		%	*n*	%	*n*	
Age categories of the women, (*n*)**															
≤ 19 years	13·52	765	13·00	1696	−0·52^a^	12·84	880	13·34	1581	0·5^a^	12·94	280	13·18	2181	0·24
20–24 years	33·97	1922	34·79	4539	0·82	34·25	2348	34·71	4113	0·46	34·44	745	34·55	5716	0·11
≥ 25 years	52·51	2971	52·21	6813	−0·3	52·92	3628	51·95	6156	−0·97	52·61	1138	52·26	8646	−0·35
Years of schooling, % (*n*)**															
No education	**43·72**	**1214**	**56·28**	**1563**	**12·56** ^**d**^	**50·85**	**1412**	**49·15**	**1365**	**−1·70** ^**d**^	**14·48**	**402**	**85·52**	**2375**	**71·04** ^**d**^
Primary incomplete	**37·63**	**1275**	**62·37**	**2123**	**24·74** ^**d**^	**43·89**	**1487**	**56·11**	**1901**	**12·22** ^**d**^	**12·87**	**436**	**87·13**	**2952**	**74·26** ^**d**^
Primary complete	**34·90**	**786**	**65·10**	**1466**	**30·2** ^**d**^	**42·27**	**952**	**57·73**	**1300**	**15·46** ^**d**^	**13·72**	**309**	**11·75**	**1943**	**−1·97** ^**d**^
Secondary incomplete	**27·06**	**1961**	**72·94**	**5285**	**45·88** ^**d**^	**33·25**	**2409**	**66·75**	**4837**	**33·50** ^**d**^	**34·54**	**747**	**86·28**	**6499**	**51·74** ^**d**^
Secondary or more	**13·87**	**422**	**86·13**	**2621**	**72·26** ^**d**^	**19·59**	**596**	**80·41**	**2447**	**60·82** ^**d**^	**8·84**	**269**	**91·16**	**2774**	**82·32** ^**d**^
Wealth index, % (*n*)**															
Poorest	**42·42**	**1875**	**57·58**	**2545**	**15·16** ^**d**^	**48·67**	**2151**	**51·33**	**2269**	**2·66** ^**d**^	**13·82**	**611**	**86·18**	**3809**	**72·36** ^**d**^
Poorer	**34·87**	**1308**	**65·13**	**2443**	**30·26** ^**d**^	**42·47**	**1953**	**57·53**	**2158**	**15·06** ^**d**^	**13·01**	**488**	**86·99**	**3263**	**73·98** ^**d**^
Middle	**30·03**	**1035**	**69·97**	**2411**	**39·94** ^**d**^	**37·09**	**1278**	**62·91**	**2168**	**25·82** ^**d**^	**11·38**	**392**	**88·54**	**3054**	**77·16** ^**d**^
Richer	**25·65**	**911**	**74·35**	**2640**	**48·70** ^**d**^	**31·12**	**1105**	**68·88**	**2446**	**37·76** ^**d**^	**10·17**	**361**	**89·83**	**3190**	**79·66** ^**d**^
Richest	**14·95**	**529**	**85·05**	**3009**	**70·10** ^**d**^	**20·60**	**729**	**79·40**	**2809**	**58·80** ^**d**^	**8·79**	**311**	**91·21**	**3227**	**82·42** ^**d**^
Number of parties, % (*n*)**															
One	**25·90**	**1534**	**74·10**	**4388**	**48·20** ^**d**^	**32·17**	**1905**	**67·83**	**4017** ^**d**^	**35·66** ^**d**^	**11·16**	**661**	**88·84**	**5261**	**77·68** ^**b**^
Two	**28·38**	**1883**	**71·62**	**4751**	**43·24** ^**d**^	**34·91**	**2316**	**65·09**	**4318**	**30·18** ^**d**^	**11·03**	**732**	**88·97**	**5902**	**77·94** ^**b**^
Three or more	**36·44**	**2241**	**63·56**	**3909**	**27·12** ^**d**^	**42·85**	**2635**	**57·15**	**3515**	**14·30** ^**d**^	**12·52**	**770**	**87·48**	**5380**	**74·96** ^**b**^
Involved in microcredit, % (*n*)***	**34·67**	**1376**	**65·17**	**2575**	**30·50** ^**d**^	**44·39**	**1754**	**55·61**	**2197** ^**d**^	**11·22** ^**d**^	**14·96**	**591**	**85·04**	**3360**	**70·08** ^**c**^
Currently working, % (*n*)***	26·12	1478	25·64	1347	−0·48^a^	25·51	1749	25·94	3074	0·43^a^	**23·25**	**503**	**26·12**	**4320**	**2·87** ^**c**^

*Student’s *t* test.

**Goodman–Kruskal gamma test.

***Chi-square test.

^a^ *P* > 0·05; ^b^ *P* < 0·05; ^c^ *P* < 0·01; ^d^ *P* < 0·001.

All bold digits referred as statistically significant (*P* < 0.05).

### Status of the factors of three domains of the Survey-Based Women’s Empowerment Index among women

Table 3 presents a comparison of fourteen variables related to the domains of the SWPER, stratified by the nutritional status of offspring. The results indicate that factors within the social independence domain, such as frequency of newspaper reading, years of education, age at first birth and age at first cohabitation, were more favourable among women with well-nourished children compared to those with undernourished children. While the median education difference between women and their husbands was 0 years, a significant distinction emerged between women with undernourished and well-nourished children (*P* < 0·001; Table 3).

**Table 3.** Comparison of the social independence, intrinsic agency and instrumental agency among women by the nutritional status of under-five children

Variables	Height-for-age Z score				Direction of difference (B-A)	Weight-for-age Z score				Direction of difference (D-C)	Weight-for-height Z score				Direction of difference (F-E)
	< −2·00 sd (A)		≥ −2·00 sd (B)			< −2·00 sd (C)		≥ −2·00 sd (D)			< −2·00 sd (E)		≥ −2·00 sd (F)		
*n*	5658		13 048			6856		11 850			5658		13 048		
	%	*n*	%	*n*		%	*n*	%	*n*		%	*n*	%	*n*	
Social independence															
Reading newspaper, % (*n*)*	8·08	457	15·84	2065	7·76^d^	24·66	622	75·34	1900	50·68^d^	9·87	249	90·13	2273	80·26^d^
	Median	IQR	Median	IQR		Median	IQR	Median	IQR		Median	IQR	Median	IQR	
Highest year of education of the women, Median (IQR)***	5	2, 7	7	4, 9	2^d^	5	2, 8	7	4, 9	2^a^	5	2, 8	6	4, 9	1^d^
	Mean	sd	Mean	sd		Mean	sd	Mean	sd		Mean	sd	Mean	sd	
Women age at first birth, Mean (sd)****	17·71	2·97	18·39	3·36	0·68^d^	17·74	3·01	18·45	3·37	0·71^d^	17·85	3·08	18·23	3·28	0·38^d^
Women’s first age of cohabitation, Mean (sd)****	15·77	2·46	16·33	2·97	0·56^d^	15·78	2·54	16·38	2·97	0·6^d^	15·89	2·73	16·20	2·84	0·31^d^
	Median	IQR	Median	IQR		Median	IQR	Median	IQR		Median	IQR	Median	IQR	
Age difference between husband and wife, Median (IQR)***	−7	6	−8	6	−1^a^	−7	6	−8	6	−1^a^	−8	6	−7	6	1^a^
Years of education difference between husband and wife, Median (IQR)**	0	4	0	4	0^d^	0	4	0	4	0^d^	0	4	0	5	0^b^
	%	*n*	%	*n*		%	*n*	%	*n*		%	*n*	%	*n*	
Intrinsic agency															
Beating is justified if the wife goes out without the husband’s permission, % (*n*)**															
Not justified	84·62	4788	88·29	11 250	3·67^d^	88·71	10 512	87·18	16 308	−1·53^d^	92·42	1999	93·62	15 488	1·20^d^
Justified	15·16	858	11·60	1523	−3·56^d^	11·15	1321	12·68	2341	1·53^d^	7·49	162	6·04	999	−1·45^d^
Do not know	0·21	12	0·11	15	−0·1^d^	0·14	17	0·14	27	0^d^	0·09	2	0·34	56	0·25^d^
Beating is justified if the wife neglects the children, % (*n*)**															
Not justified	82·80	4680	85·71	11 172	2·91^d^	82·72	5667	86·06	10 185	3·34^d^	94·64	2047	96·89	16 028	2·25^d^
Justified	16·99	960	14·17	1847	−2·82^d^	17·14	1174	13·80	1633	−3·34^d^	5·09	110	2·93	485	−2·16^d^
Do not know	0·21	12	0·21	15	0^d^	0·15	10	0·14	17	−0·01^d^	0·28	6	0·18	30	−0·10^d^
Beating is justified if the wife argues with husband, % (*n*)**															
Not justified	77·67	4394	82·17	10 722	4·5^d^	78·03	5349	82·42	9767	4·39^d^	36·68	793	34·14	5647	−2·54^a^
Justified	22·15	1253	17·67	2306	−4·48^d^	21·75	1491	17·45	2068	−4·30^d^	55·18	1193	58·45	9668	3·27^a^
Do not know	0·18	10	0·15	20	−0·03^d^	0·22	15	0·13	15	−0·09^d^	8·14	176	7·41	1226	−0·73^a^
Beating is justified if the wife refuses to have sex with husband, % (*n*)**															
Not justified	91·62	5184	94·29	12 303	2·67^d^	91·48	6272	94·64	11 215	3·16^d^	92·42	1999	93·62	15 488	1·20^c^
Justified	8·01	453	5·43	708	−2·58^d^	8·10	555	5·11	606	−2·99^d^	7·49	162	6·04	999	−1·45^c^
Do not know	0·37	21	0·28	37	−0·09^d^	0·42	29	0·24	29	−0·18^d^	0·09	2	0·34	56	0·25^c^
Beating is justified if the wife burns food, % (*n*)**															
Not justified	95·96	5412	97·05	12 663	1·09^d^	95·46	6545	97·30	11 530	1·84^d^	94·64	2047	96·89	16 028	2·25^d^
Justified	4·21	238	2·74	357	−1·47^d^	4·32	296	2·52	299	−1·8^d^	5·09	110	2·93	485	−2·16^d^
Do not know	0·114	8	0·21	28	0·096^d^	0·22	15	0·18	921	−0·04^d^	0·28	6	0·18	30	−0·1^d^
Instrumental agency															
A person who usually decides on respondent’s healthcare, % (*n*)**															
Husband or other family member	36·65	2073	33·47	4367	−3·18^d^	36·39	2494	33·30	3946	−3·09^d^	36·68	793	34·14	5647	−2·54^b^
Jointly with husband	55·92	3163	59·00	7698	3·08^d^	55·73	3819	59·43	7042	3·7^d^	55·18	1193	58·45	9668	3·27^b^
Woman herself	7·63	420	7·53	982	−0·1^d^	7·88	540	7·27	862	−0·61^d^	8·14	176	7·41	1226	−0·73^b^
Person who usually decides on large household purchase, % (*n*)**															
Husband or other family member	40·64	2299	38·24	4989	−2·4^c^	40·71	2791	37·95	4497	−2·76^c^	41·38	895	38·65	6393	−2·73^b^
Jointly with husband	55·75	3154	58·35	7613	2·6^c^	55·78	3824	58·60	6943	2·82^c^	54·69	1183	57·94	9584	3·25^b^
Woman herself	3·61	204	3·41	445	−0·2^c^	3·50	240	3·54	409	0·04^c^	3·93	85	3·41	564	−0·52^b^
Person who usually decides on visiting family or relatives, % (*n*)**															
Husband or other family member	38·37	2170	35·55	4636	−2·82^c^	39·28	2692	34·73	4114	−4·55^d^	39·81	861	35·96	5945	−3·85^d^
Jointly with husband	56·70	3207	59·05	7701	2·35^c^	55·41	3797	60·04	7111	4·63^d^	54·65	1182	58·82	9726	4·17^d^
Woman herself	4·93	279	5·40	704	0·47^c^	5·31	367	5·23	616	−0·08^d^	5·55	120	5·22	863	−0·33^d^

IQR, interquartile range.

*Chi-square test.

**Goodman–Kruskal gamma test.

***Kruskal–Wallis rank test.

****Student’s *t* test.

^a^ *P* > 0·05; ^b^ *P* < 0·05; ^c^ *P* < 0·01; ^d^ *P* < 0·001.

We found that a higher proportion of women with well-nourished children expressed disagreement with the rationale behind five specific justifications for IPV. Additionally, our findings revealed that women had limited autonomy in individual decision-making regarding their healthcare, important household expenditures and visits to family and relatives across all groups, with most decisions being made jointly with their husbands (Table 3).

### Prevalence of acute respiratory tract infection and receiving treatment from medically trained providers

The prevalence of ARI among undernourished children was higher than that of well-nourished children (*P* < 0·05; Appendix 1). Additionally, a greater proportion of well-nourished children suffering from ARI in the 2 weeks preceding the interview received treatment from MTP compared to those who were undernourished (Appendix 1). We observed that the prevalence of ARI displayed an inverse relationship with factors related to social independence, intrinsic agency and instrumental agency (Appendix 3).

### Pathways between the domains of Survey-Based Women’s Empowerment Index, height-for-age Z score, food intake of under-five children and access to healthcare services

**Figure 2.**
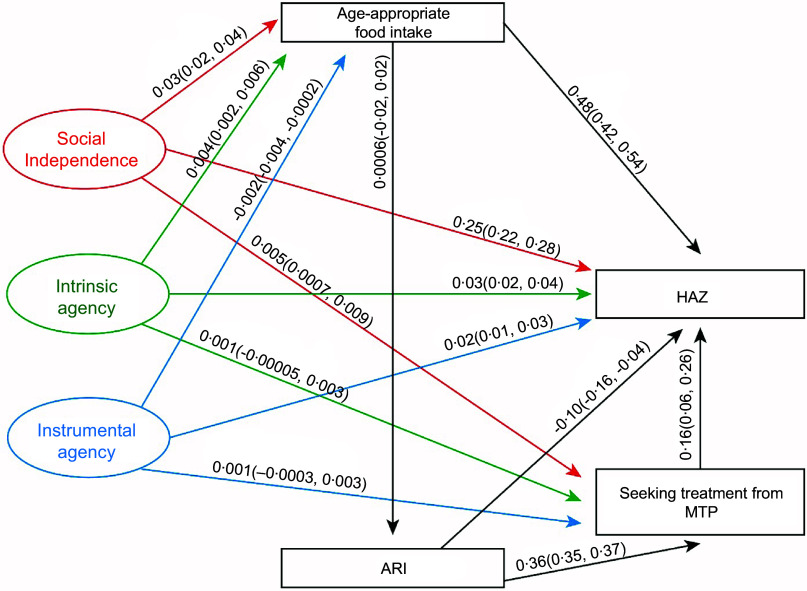
Pathways between women’s social independence, intrinsic and instrumental agency and improved height-for-age Z score of under-five children. HAZ, height-for-age Z score; ARI, acute respiratory tract infection; MTP, medically trained providers.

Figure 2 presents that social independence, intrinsic agency and instrumental agency had significant positive effects on children’s HAZ scores, with estimated coefficients of 0·25 (95 % CI: 0·22, 0·28), 0·03 (0·02, 0·04) and 0·02 (0·01, 0·03), respectively. We observed similar effects of SWPER domains on HAZ in the segregated data (Appendix 2). Additionally, both social independence and intrinsic agency were positively associated with age-appropriate food intake among under-five children, with coefficients of 0·03 (0·02, 0·04) and 0·004 (0·002, 0·006), respectively. In contrast, instrumental agency negatively influenced age-appropriate food intake (–0·002 (–0·004, –0·0002)). Moreover, social independence also positively affected care-seeking from MTP at the onset of ARI (0·005 (0·0007, 0·009)).

### Pathways between the domains of Survey-Based Women’s Empowerment Index, weight-for-age Z score, food intake of under-five children and access to healthcare services

**Figure 3.**
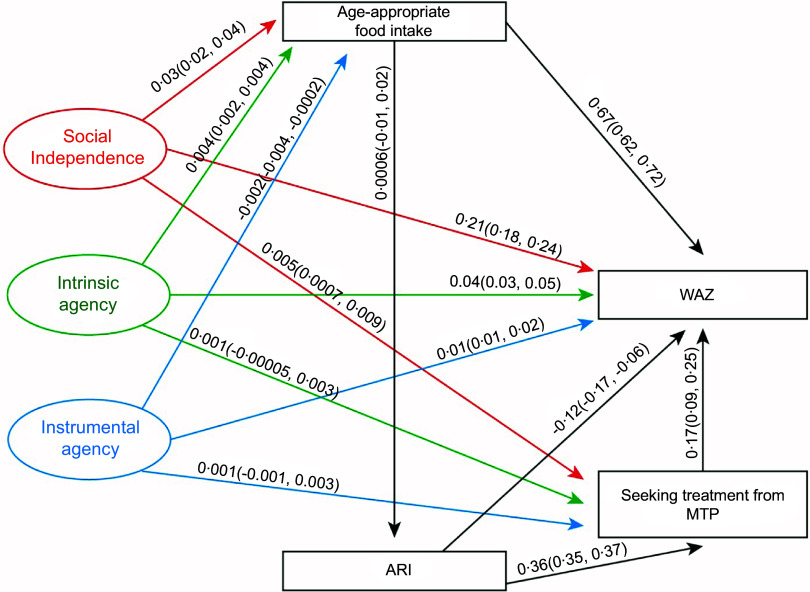
Pathways between women’s social independence, intrinsic and instrumental agency and improved weight-for-age Z score of under-five children. WAZ, weight-for-age Z score; ARI, acute respiratory tract infection; MTP, medically trained providers.

Figure 3 shows the effects of the three domains of the SWPER on the WAZ score and its underlying determinants. The results indicated that social independence, intrinsic agency and instrumental agency had significant positive associations with improved WAZ scores, with estimated coefficients of 0·21 (0·18, 0·24), 0·04 (0·03, 0·05) and 0·01 (0·01, 0·02), respectively. We found similar effects of the SWPER domains of the WAZ in the segregated data (Appendix 2). Additionally, both social independence and intrinsic agency domains were positively associated with age-appropriate food intake, with effect sizes of 0·03 (0·02, 0·04) and 0·004 (0·002, 0·004), respectively. In contrast, instrumental agency had a small but significant negative association with food consumption among children (–0·002 (–0·004, –0·0002)). Furthermore, only social independence was positively associated with seeking treatment from MTP for ARI, with an estimated effect of 0·005 (0·0007, 0·009).

### Pathways between the domains of Survey-Based Women’s Empowerment Index, weight-for-height Z score, food intake of under-five children and access to healthcare services

**Figure 4.**
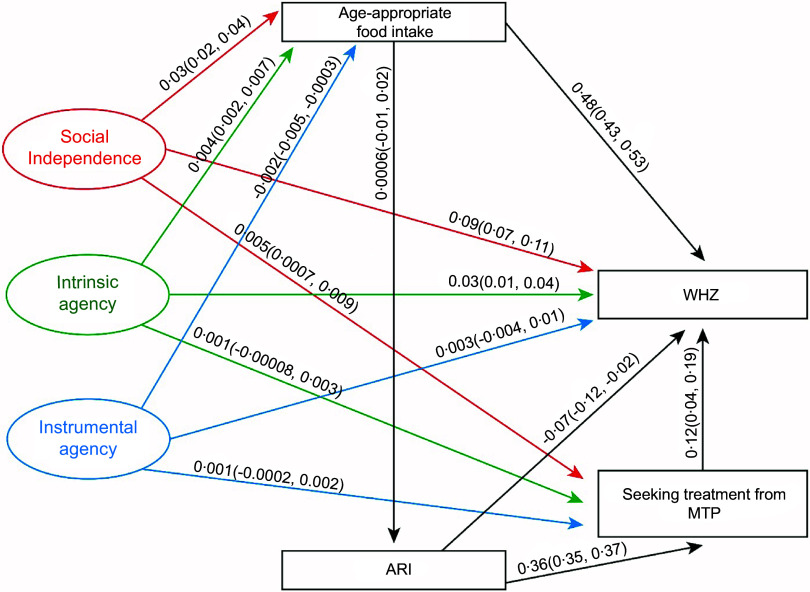
Pathways between women’s resources and agency and improved weight-for-height Z score of under-five children suffering from ARI in Bangladesh. WHZ, weight-for-height Z score; ARI, acute respiratory tract infection; MTP, medically trained providers.

We conducted a separate GSEM to examine the effects of the three SWPER domains on WHZ scores among children under 5 years of age (Figure 4). Maternal social independence and intrinsic agency were both positively associated with WHZ, with effect sizes of 0·09 (0·07, 0·12) and 0·03 (0·02, 0·04), respectively. We observed similar effects during 2014 and 2017–2018, however, not during 2011 (Appendix 2). These two domains also had positive effects on age-appropriate food intake: 0·03 (0·02, 0·04) for social independence and 0·005 (0·003, 0·007) for intrinsic agency. In contrast, instrumental agency was negatively associated with age-appropriate food intake, with an effect size of –0·003 (–0·005, –0·0003). Additionally, social independence positively influenced care-seeking from MTP at the onset of ARI, with an effect size of 0·005 (0·001, 0·01).

### Pathways between age-appropriate food intake and nutritional status of under-five children

Figures 2–4 demonstrate that age-appropriate food intake was positively associated with all three nutritional indicators among offspring. Its effect was strongest on the WAZ score (0·67 (0·62, 0·72); Figure 3), compared to the HAZ score (0·48 (0·42, 0·54); Figure 2) and WHZ score (0·48 (0·43, 0·53); Figure 4).

### Pathways between acute respiratory tract infection and seeking treatment from medically trained providers and nutritional status of under-five children

We observed that the recent episode of ARI deteriorated the HAZ score (–0·10 (–0·16, –0·04)); in contrast, children who received treatment from MTP had a better HAZ- score (0·16 (0·06, 0·26)) (Figure 2). Furthermore, the prevalence of ARI among under-five children led to women seeking treatment from MTP for ARI (0·36 (0·35, 0·37)).

Additionally, Figure 3 showed that the recent episodes of ARI among children had a detrimental effect on WAZ (–0·12 (–0·17, –0·06)). However, it increased women’s mobility to MTP for treatment (0·36 (0·35, 0·37)). Importantly, treatment from MTP had a beneficial impact on the WAZ score (0·17 (0·09, 0·25)).

Similarly, Figure 4 revealed that the recent episodes of ARI among children had a positive effect on seeking treatment from MTP (0·37 (0·36, 0·38)) and a negative effect on the WHZ score (–0·07 (–0·12, –0·21)), and treatment from MTP improved WHZ with a coefficient of 0·12 (0·04, 0·20) (Figure 4).

### Mediation analysis

We performed mediation analyses to explore which mediators link the three domains of the SWPER to HAZ, WAZ and WHZ scores (Table 4). Our findings revealed significant indirect effects of social independence and intrinsic agency on HAZ and WAZ in the presence of age-appropriate food intake (Table 4). Additionally, results showed that seeking treatment from MTP at the onset of ARI also acted as a mediator on the pathways connecting social independence, intrinsic agency and instrumental agency with HAZ, WAZ and WHZ scores (Table 4).

**Table 4.** Direct, indirect and total effect of social independence, intrinsic agency and instrumental agency on HAZ, WAZ and WHZ scores of under-five children

	Direct effect		Indirect effect		Total effect			
Model	Coefficient	95 % CI	Coefficient	95 % CI	Coefficient	95 % CI	Mediator	Mediation
HAZ								
SI-> HAZ	1·91	1·74, 2·09	**1·01**	**0·80, 1·23**	2·93	2·66, 3·20	Children’s diet	Yes
SI-> Diet								
Diet-> HAZ								
InA-> HAZ	0·23	0·18, 0·27	**0·16**	**0·10, 0·22**	0·39	0·31, 0·47	Children’s diet	Yes
InA-> Diet								
Diet-> HAZ								
InsA-> HAZ	0·20	0·14, 0·26	−0·02	–0·10, 0·04	0·17	0·07, 0·26	Children’s diet	No
InsA-> Diet								
Diet-> HAZ								
SI-> HAZ	2·11	1·92, 2·30	**0·06**	**0·002, 0·12**	2·17	1·97, 2·37	ARI_MTP	Yes
SI-> ARI MTP								
ARI MTP-> HAZ								
InA-> HAZ	0·21	0·11, 0·30	**0·08**	**0·03, 0·13**	0·29	0·18, 0·40	ARI_MTP	Yes
InA-> ARI MTP								
ARI MTP-> HAZ								
InsA-> HAZ	0·07	–0·06, 0·02	**0·08**	**0·01, 0·14**	0·15	0·005, 0·30	ARI_MTP	Yes
InsA-> ARI MTP								
ARI MTP-> HAZ								
WAZ								
SI-> WAZ	1·73	1·58, 1·89	**1·43**	**1·15, 1·70**	3·16	2·85, 3·48	Children’s diet	Yes
SI-> Diet								
Diet-> WAZ								
InA-> WAZ	0·24	0·20, 0·28	**0·22**	**0·14, 0·30**	0·46	0·37, 0·56	Children’s diet	Yes
InA -> Diet								
Diet> WAZ								
InsA-> WAZ	0·18	0·13, 0·24	−0·04	–0·14, 0·06	0·14	0·03, 0·26	Children’s diet	No
InsA-> Diet								
Diet-> WAZ								
SI-> WAZ	1·79	1·42, 2·16	**0·55**	**0·31, 0·79**	2·34	1·92, 2·76	ARI_MTP	Yes
SI-> ARI_MTP								
ARI_MTP-> WAZ								
InA-> WAZ	0·20	0·12, 0·29	**0·08**	**0·03, 0·13**	0·29(0·19, 0·39)		ARI_MTP	Yes
InA -> ARI_MTP								
ARI_MTP-> WAZ								
InsA-> WAZ	0·14	0·02, 0·26	**0·08**	**0·02, 0·14**	0·22	0·08, 0·36	ARI_MTP	Yes
InsA -> ARI_MTP								
ARI_MTP-> WAZ								
WHZ								
SI-> WHZ	0·86	0·72, 1·01	**1·02**	**0·81, 1·23**	1·89	1·64, 2·14	Children’s diet	Yes
SI-> Diet								
Diet-> WHZ								
InA-> WHZ	0·15	0·11, 0·19	**0·15**	**0·09, 0·21**	0·31	0·24, 0·38	Children’s diet	Yes
InA-> Diet								
Diet-> WHZ								
InsA-> WHZ	0·08	0·03, 0·13	−0·02	–0·010, 0·04	0·05	–0·02, 0·14	Children’s diet	No
InsA-> Diet								
Diet-> H WHZ								
SI-> WHZ	0·80	0·45, 1·15	**0·36**	**0·16, 0·56**	1·66	0·78, 1·54	ARI_MTP	Yes
SI-> ARI_MTP								
ARI_MTP-> WHZ								
InA-> WHZ	0·11	0·03, 0·19	**0·05**	**0·01, 0·08**	0·16	0·07, 0·25	ARI_MTP	Yes
InA -> ARI_MTP								
ARI_MTP-> WHZ								
InsA-> WHZ	0·09	–0·01, 0·21	**0·05**	**0·008, 0·09**	0·14	0·02, 0·26	ARI_MTP	Yes
InsA -> ARI_MTP								
ARI_MTP-> WHZ								

HAZ, height-for-age Z score; WAZ, weight-for-age Z score; WHZ, weight-for-height Z score; SI, social independence; InA, intrinsic agency; InsA, instrumental agency; ARI, acute respiratory tract infection; MTP, medically trained providers.

Children’s diet – age-appropriate diet intake by the under-five children during the last 24 h preceding the interview.

ARI_MTP – received treatment from medically trained providers on set of ARI during the last 2 weeks preceding the interview.

All bold digits referred as statistically significant (*P* < 0.05).

## Discussion

This research revealed significant insights into the impact of women’s social independence and intrinsic agency on improving the nutritional status of offspring, while instrumental agency has a smaller effect on HAZ. Findings revealed that these domains not only directly enhanced the nutritional status of under-five children but also indirectly through promoting appropriate food consumption and access to healthcare at the commencement of ARI. The study identified that treatment for ARI could prevent undernutrition in under-five children, serving as a crucial mediator for all domains of women’s empowerment in improving nutritional status. Additionally, it highlighted that women in Bangladesh face a severe lack of autonomy in decision-making, which adversely affects these children’s dietary intake. These findings underscore the crucial connections between SDG-2, striving for zero hunger, SDG-3, advocating for a healthy life, and SDG-5, which highlights gender equality.

This study highlighted the critical role of women’s social independence in improving nutritional status, emphasising the need to prioritise factors that enhance this domain for greater impact on nutrition. We observed that each element linked to women’s social independence – such as newspaper reading, years of schooling, age at first birth, age at first cohabitation and education differences between spouses – independently contributed to the nutritional well-being of Bangladeshi children. Moreover, our research linked the pathways that social independence was key in improving underlying factors of improved nutritional status, such as age-appropriate food intake and timely treatment for ARI, aligning with UNICEF’s ‘*food-health-care*’ model^(30)^. Additionally, social independence was also found effective for improving the nutritional status of Asian and African children through the pathways by enhancing intrinsic and instrumental agency^(18)^. In contrast, social independence, intrinsic agency and instrumental agency were positively associated with the nutritional status of offspring in Gambia, even after adjusting for diet and care adjusted as confounders which meant that domains of SWPER independently could improve the nutritional status of under-five children^(8)^. However, the roles of appropriate food consumption and care-seeking during illness are crucial for preventing undernutrition.

We observed that the effect of intrinsic agency on nutritional outcomes was not consistent. Although most women stated IPV was unjustifiable for any of the five given reasons, this might reflect a potential response bias or a heightened sense of empowerment among respondents. Ensuring privacy during such data collection is crucial, as the absence of privacy can greatly increase the likelihood of response bias. Moreover, Bangladesh is one of nineteen countries with the highest rates of IPV, with partnered women aged 15–49 years experiencing IPV at varying rates across regions due to social norms^(31,32)^. Key factors contributing to IPV in Bangladesh include early marriage, young age, low education, poverty, exposure to maternal abuse, childhood violence, dowry and poor spousal communication^(33)^. Since we lack data on the prevalence of IPV in the current cohort, we cannot measure the correlation between IPV prevalence and factors associated with women’s intrinsic agency. Research showed that women who consider wife-beating ‘unjustifiable’ were more likely to recognise their increased sense of entitlement, self-esteem, self-efficacy and status, which positively reflects their sense of empowerment, while strong intrinsic agency is expressed through decisional-making autonomy as a key component of instrumental agency^(34,35)^. Our observations indicated that while Bangladeshi women felt empowered, this did not effectively translate into greater autonomy of decision-making in accessing food security and healthcare, contributing to undernutrition in their under-five children. Other factors, such as teenage marriage, pregnancy and IPV, may cause reproductive health issues, leading to low birth weight and short stature later in life^(36)^.

Furthermore, intrinsic agency did not improve women’s autonomy in seeking treatment for ARI. These women may have had a knowledge gap regarding the critical health issues of ARI and the necessity for treatment. Improved attitudes towards the unjustifiability of wife-beating are associated with factors such as educational empowerment, access to media and decision-making autonomy^(37)^. Consequently, the minimal instrumental agency observed in these women might be a lower impact of self-efficacy, which is an important component of intrinsic agency^(35)^. Moreover, IPV often led to depression and post-traumatic stress disorders in women, which could diminish their inclination to provide adequate care for their children’s health and nutrition^(38)^.

Our results revealed that women had limited autonomy in important household decision-making, which undermined their instrumental agency in providing healthy diets and improving nutritional status for their children. Many women in this study lack the independence to make decisions and often rely on spouses or family members for healthcare. As primary caregivers, the quality of care they provide is a critical determinant of nutritional status, alongside food security and environmental health factors^(30,39)^. The patriarchal social context in Bangladesh, compounded by cultural beliefs, restricts women’s control over household resources, limits their freedom of movement and hinders their decision-making for their own well-being and that of their children. These challenges mirror those faced in other developing countries^(12,13,40)^.

We observed pathways where ARI affects underweight and wasting of offspring, consistent with other studies showing that ARI has an immediate impact on children’s weight^(41)^. Children suffering from ARI may also have experienced diarrhoea in the preceding 2 weeks, which can exacerbate underlying moderate or severe malnutrition and further impair immunity to pneumonia^(41)^. Our data aligned with clinical studies indicating that children admitted with ARI symptoms often suffer from various forms of undernutrition, such as anaemia, stunting and wasting^(4,23,42)^. This vulnerable subgroup faces an elevated mortality risk due to severe acute malnutrition and treatment failure, which can be mitigated through timely hospitalisation^(43)^.

We identified a unique pathway in which utilising healthcare services from MTP at the onset of ARI can improve the nutritional status of under-five children, particularly regarding stunting and underweight, and act as a mediator between women’s empowerment and nutritional outcomes. Timely treatment enhances immunity and halts further deterioration into undernutrition. Unfortunately, there is a significant inequity in care-seeking between boys and girls with ARI in Bangladesh, with 46 % of boys receiving care compared to only 31 % of girls^(24)^. Our study indicates that social independence may enhance the utilisation of treatment from MTP, likely due to associations with maternal education and media exposure. In contrast, parental ignorance about ARI symptoms, low-quality care at facilities and poverty hinder access to MTP, particularly for women with limited social independence and autonomy to seek treatment for their children^(21,44,45)^.

We also observed that not all women had access to health facilities, even when their children were ill. The government of Bangladesh has adopted the IMCI strategy at primary, secondary and tertiary levels to progress towards the Global Action Plan for Pneumonia and Diarrhea, aiming to prevent all avoidable pneumonia-related deaths by 2025^(21,24,46)^. Additionally, the national vaccination programme, which includes the Haemophilus influenzae type b vaccine, is helping to reduce pneumonia-related mortality in Bangladesh^(47)^. However, policies alone will not improve the situation; awareness must be enhanced among both men and women in Bangladesh to access resources and opportunities provided by the government^(48)^.

Most importantly, we found that women’s empowerment not only improved children’s nutritional status but also enhanced dietary intake and access to MTP at the onset of ARI. These two indicators serve as mediators in improving children’s nutritional outcomes. Therefore, integrated approaches are needed in Bangladesh to empower women and address their knowledge gap regarding age-appropriate food intake and ARI treatment, as outlined in UNICEF’s conceptual framework for addressing undernutrition.

Finally, we observed that the three domains of the SWPER index did not contribute equally to improve the nutritional outcomes of children. Among these, social independence demonstrated the most substantial impact, particularly on HAZ, followed by WAZ and WHZ scores. The HAZ score, a critical indicator of chronic undernutrition, is primarily influenced during the ‘first 1000 d’^(49)^. During this period, adequate maternal and child nutrition is crucial to prevent stunting, as growth faltering during this window is largely irreversible(1). WAZ may be affected by moderate food insecurity and infectious diseases, while WHZ reflects acute malnutrition, often driven by severe poverty, illness and food deprivation^(50)^. The domain of social independence includes indicators such as women’s age at marriage, level of education, spousal age and education gaps and exposure to mass media. These characteristics are typically better among women from affluent families, where food insecurity is less common. Such women in Bangladesh are more likely to receive appropriate care during pregnancy, childbirth and the postpartum period. Consequently, their children are more likely to consume age-appropriate food, indirectly leading to improved nutritional outcomes – findings that are strongly supported by our analysis.

In contrast, gender inequality remains more pronounced in lower socio-economic groups, where poor household structure, higher religious conservatism, illiteracy and male dominance constrain women’s autonomy in decision-making. These factors diminish women’s sense of empowerment and restrict their ability to make strategic life choices, particularly those affecting their children’s health and nutrition. Our results also indicated minimal or no effects of intrinsic and instrumental agency on child nutritional outcomes. This suggests a general lack of ‘power within’ and ‘power to’ among Bangladeshi women, regardless of socio-economic status^(51)^. Furthermore, trends observed across survey years reflect a persistent causal link between limited women’s empowerment and poor child nutrition. This underscores the urgent need to strengthen women’s self-awareness and decision-making autonomy as a pathway to improve the nutritional status of their children.

### Policy implication

The National Women Development Policy of Bangladesh (2011) outlines twenty-two objectives, six of which align with this study: women’s educational and socio-economic development, necessary support services, economic empowerment, prevention of violence against women and girls and women’s access to mass media^(52)^. This policy also includes the Child Marriage Restraint Act (2017) to prevent child marriage in Bangladesh^(53)^. While the policy aims to promote gender equality in various sectors, it lacks specific provisions for enhancing women’s decision-making capacity at the micro level. To effectively address children suffering from ARI, the government must strengthen awareness raising among parents through campaigns, community engagement and mass media and monitor implementation of the IMCI strategy. Education and economic empowerment are fundamental to improving all three domains of women’s empowerment. We urge the government of Bangladesh, Non-Governmental Organization and development partners to take necessary measures to tackle these issues.

### Limitations

There are some limitations in this study. First, though there were negative correlations between distinct domains of women’s empowerment and ARI, we could not build the pathways among them due to non-convergence issues during analysis (Appendix 3). We believe that due to a lack of components of intrinsic agency such as self-efficacy and personal initiative, women could not choose effective measures for preventing ARI. Therefore, we cannot conclude that these domains have no effect on ARI prevalence. A case–control study may better establish the association between empowerment domains and ARI incidence. Second, we analysed the last 2 weeks of recall data to establish the pathways between ARI and undernutrition, and there is a lack of data on earlier episodes of diarrhoea; therefore, we cannot explain the reason for the drastic weight loss of the children, which is reflected by the indicators underweight and wasting. Third, to avoid the complexity of the analysis, we did not adjust sociodemographic variables within the pathways. The SWPER metric measures empowerment of only partnered women but not the women who are divorced, widowed and single. Thus, findings of the study cannot be generalised with these categories of women. More limitations on this matrix published elsewhere^(26)^. Finally, the estimated goodness of fit, Akaike information criterion or Bayesian information criterion values for each of the GSEM models indicated that the models have a good fit with a balanced trade-off between fit and complexity. Since we did not build simpler models for each of the outcome indicators, we are unable to conclusively assess the goodness of fit of the GSEM models.

### Conclusion

In conclusion, this study highlights the importance of addressing factors related to social independence and intrinsic agency of women for sustainable improvement in the nutrition outcomes of under-five children in Bangladesh. Scaling up interventions that focus on these aspects is crucial for achieving better nutritional status of under-five children in Bangladesh and similar contexts.

## Supporting information

Jolly et al. supplementary material 1 Jolly et al. supplementary material

Jolly et al. supplementary material 2 Jolly et al. supplementary material

## Supplementary material

For supplementary material accompanying this paper visit https://doi.org/10.1017/S1368980026101980
