# Supplementary material for: Women’s empowerment and nutritional status of children: new evidence for Bangladesh
Source: Public Health Nutr. 2026 Feb 18;29(1):e46. doi: 10.1017/S1368980026101980 (PMC13087980; doi:10.1017/S1368980026101980)
Supplement: Jolly et al. supplementary material 2 — Jolly et al. supplementary material [file S1368980026101980sup002.docx]

Appendix

**Appendix 2: Effect of latent and exogenous variables on endogenous variables**

| **Endogenous variable** | **Latent/exogenous variable** | **Co-efficient (95% confidence interval** | | |
| --- | --- | --- | --- | --- |
|  |  | **Year 2011** | **Year 2014** | **Year 2017-2018** |
| **HAZ** | Social independence | **0.26 (0.20, 0.31)*** | **0.23(0.18, 0.28)*** | **0.24(0.21,0.27)*** |
|  | Intrinsic Agency | **0.02 (0.007, 0.03)*** | **0.03(0.01, 0.05)*** | **0.03(0.02, 0.04)*** |
|  | Instrumental Agency | **0.03 (0.009, 0.04)*** | **0.02(0.001, 0.03)*** | **0.02(0.01,0.03)*** |
|  | Age-appropriate food intake | **0.57(0.46, 0.68)*** | **0.62(0.51, 0.73)*** | **0.048(0.42,0.54)*** |
|  | Seeking treatment from MTPs | **0.20(0.04, 0.37)*** | **0.19(0.008,0.35)*** | **0.16(0.06,0.26)*** |
|  | ARI | **-0.12(-0.21, -0.01)*** | **-0.13(-0.23, -0.02)*** | **-0.01(-0.16, -0.04)*** |
|  |  |  |  |  |
| **WAZ** | Social independence | **0.21(0.16, 0.25)*** | **0.21(0.17, 0.25)*** | **0.21(0.18,0.23)*** |
|  | Intrinsic Agency | **0.03(0.01, 0.04)*** | **0.04(0.02,0.05)*** | **0.04(0.03,0.05)*** |
|  | Instrumental Agency | **0.02(0.002, 0.03)*** | **0.01(0.001,0.03)*** | **0.01(0.007, 0.02)*** |
|  | Age-appropriate food intake | **0.79 (0.70, 0.88)*** | **0.71(0.61,0.80)*** | **0.67(0.62, 0.73)*** |
|  | Seeking treatment from MTPs | **0.27 (0.14, 0.40)*** | 0.14(-0.009,0.28) | **0.17(0.09, 0.26)*** |
|  | ARI | **-0.14(-0.22, -0.05)*** | **-0.14(0.23, -0.04)*** | **-0.12(-0.17, -0.06)*** |
|  |  |  |  |  |
| **WHZ** | Social independence | **0.05(0.02,0.08)*** | **0.11(0.07,0.15)*** | **0.03(0.03,0.04)*** |
|  | Intrinsic Agency | 0.03(-0.005, 0.69) | **0.02(0.01,0.04)*** | **0.03(0.02,0.04)*** |
|  | Instrumental Agency | -0.003(-0.03,0.03) | 0.005(-0.007,0.02) | 0.004(-0.004,0.01) |
|  | Age-appropriate food intake | **0.59(0.50, 0.69)*** | **0.40 (0.31, 0.49)** | **0.48(0.43,0.53)*** |
|  | Seeking treatment from MTPs | **0.24(0.10, 0.38)*** | 0.06(-0.07, 0.20) | **0.12(0.04,0.12)*** |
|  | ARI | **-0.09(-0.18, -0.01)*** | **-0.09 (-0.18, -0.002)** | **-0.07(-0.12, -0.02)*** |
|  |  |  |  |  |
| **Age-appropriate food intake** | Social independence | **0.03(0.02, 0.04)*** | **0.03(0.02,0.04)*** | **0.03(0.02, 0.04)*** |
|  | Intrinsic Agency | 0.002(-0.001, 0.005) | **0.005(0.001,0.009)*** | **0.004(0.002, 0.006)*** |
|  | Instrumental Agency | **-0.001(-0.005, 0.003)*** | **-0.005(-0.008, -0.001)*** | **-0.002(-0.005,-0.0002)*** |
|  |  |  |  |  |
| **Seeking treatment from MTPs** | Social independence | **0.008 (0.00, 0.02)*** | **0.008(0.001,0.02)*** | **0.005(0.0007, 0.009)*** |
|  | Intrinsic Agency | **0.003(0.00, 0.005)*** | 0.001(-0.002,0.004) | 0.001(-0.00004, 0.002) |
|  | Instrumental Agency | **0.003(0.00, 0.006)*** | 0.001(-0.001,0.004) | 0.001(-0.0002,0.002) |
|  | ARI | **0.33(0.32,0.35)*** | **0.38(0.36,0.39)*** | **0.36(0.35, 0.37)*** |
|  |  |  |  |  |
| **ARI** | Age-appropriate food intake | 0.11(-0.09,0.32) | **-0.01(-0.25, -0.22)*** | -0.035 (-0.16, 0.09) |
|  |  |  |  |  |

HAZ- height-for-age z score; WAZ- Weight-for-age z score; WHZ-Weight-for-height z score; MTP- Medically trained providers; ARI-Acute respiratory tract infection

**P<0.05*

**Appendix 3:** Correlation between ARI among under-five children and factors of social-independence, intrinsic agency and instrumental agency of women

| **Social independence** | | | | | | | |
| --- | --- | --- | --- | --- | --- | --- | --- |
| **Variables** | ARI | Reading newspaper | Highest year of education of the women | Women age at first birth | Women's first age of cohabitation | Age difference between husband and wife | Years of education difference between husband and wife |
| ARI | 1.00 |  |  |  |  |  |  |
| Reading newspaper, | **-0.02^b^** | 1.00 |  |  |  |  |  |
| Highest year of education of the women | **-0.05^c^** | **0.43^c^** | 1.00 |  |  |  |  |
| Women age at first birth | **-0.05^c^** | **0.25^c^** | **0.36^c^** | 1.00 |  |  |  |
| Women's first age of cohabitation | **-0.05^c^** | **0.26^c^** | **0.40^c^** | **0.77^c^** | 1.00 |  |  |
| Age difference between husband and wife | 0.006 | 0.01 | **0.05^c^** | **0.11^c^** | **0.10^c^** | 1.00 |  |
| Years of education difference between husband and wife | 0.01 | **-0.04^c^** | **0.15^c^** | **-0.05^c^** | **0.05^c^** | **0.05^c^** | 1.00 |
| **Intrinsic agency** | | | | | | | |
| **Variables** | ARI | Beating is justified if the wife goes out without husband's permission | Beating is justified if the wife neglects the children | Beating is justified if wife refuses to have sex with husband | Beating is justified if the wife burn food | Beating is justified if wife argues with husband |  |
| ARI | 1.00 |  |  |  |  |  |  |
| Beating is justified if the wife goes out without husband's permission | **-0.02^c^** | 1.00 |  |  |  |  |  |
| Beating is justified if the wife neglects the children | **-0.03^c^** | **0.53^c^** | 1.00 |  |  |  |  |
| Beating is justified if wife refuses to have sex with husband | **-0.04^c^** | **0.47^c^** | 0.50^c^ | 1.00 |  |  |  |
| Beating is justified if the wife burn food | -0.005 | **0.36^c^** | **0.35^c^** | **0.40^c^** | 1.00 |  |  |
| Beating is justified if wife argues with husband | **-0.02^b^** | **0.31^c^** | **0.30^c^** | **0.29^c^** | **0.45^c^** | 1.00 |  |
| **Instrumental agency** | | | | | | | |
| **Variables** | ARI | Women decide on her healthcare | Women decide on large household purchase | Women decide on visiting family or relatives |  |  |  |
| ARI | 1.00 |  |  |  |  |  |  |
| Women decide on her healthcare | **-0.02^a^** | 1.00 |  |  |  |  |  |
| Women decide on large household purchase | **-0.02^a^** | **0.44^c^** | 1.00 |  |  |  |  |
| Women decide on visiting family or relatives | **-0.01^a^** | **0.43^c^** | **0.56^b^** | 1.00 |  |  |  |

a=*P>0.05; b=P<0.01; c=P<0.001*
